# Paralogous Ribosomal Protein L32-1 and L32-2 in Fission Yeast May Function Distinctively in Cellular Proliferation and Quiescence by Changing the Ratio of Rpl32 Paralogs

**DOI:** 10.1371/journal.pone.0060689

**Published:** 2013-04-05

**Authors:** Lei Sun, Xiaowei Yang, Feifei Chen, Rongpeng Li, Xuesong Li, Zhenxing Liu, Yuyu Gu, Xiaoyan Gong, Zhonghua Liu, Hua Wei, Ying Huang, Sheng Yuan

**Affiliations:** Jiangsu Key Laboratory for Microbes and Functional Genomics, Jiangsu Engineering and Technology Research Center for Industrialization of Microbial Resources, College of Life Science, Nanjing Normal University, Nanjing, Jiangsu, People’s Republic of China; University College London, United Kingdom

## Abstract

Fission yeast cells express Rpl32-2 highly while Rpl32-1 lowly in log phase; in contrast, expression of Rpl32-1 raises and reaches a peak level while Rpl32-2 is downregulated to a low basic level when cells enter into stationary phase. Overexpression of Rpl32-1 inhibits cell growth while overexpression of Rpl32-2 does not. Deleting *rpl32-2* impairs cell growth more severely than deleting *rpl32-1* does. Cell growth impaired by deleting either paralog can be rescued completely by reintroducing *rpl32-2*, but only partly by *rpl32-1.* Overexpression of Rpl32-1 inhibits cell division, yielding 4c DNA and multiple septa, while overexpressed Rpl32-2 promotes it. Transcriptomics analysis proved that Rpl32 paralogs regulate expression of a subset of genes related with cell division and stress response in a distinctive way. This functional difference of the two paralogs is due to their difference of 95^th^ amino acid residue. The significance of a competitive inhibition between Rpl32 paralogs on their expression is discussed.

## Introduction

Paralogous genes exist after the gene duplication event and usually code for proteins with similar function and/or structure. Gene duplication is thought to supply raw genetic material, allowing functional divergence and rapid biological evolution [1]–[3]. In yeast, most of cytoplasmic ribosomal proteins are retained in duplication. Fission yeast *Schizasaccharomyces pombe* has 80 different ribosomal proteins encoded by 143 different genes, 56 of which are encoded by two or more duplicated genes (http://ribosome.med.miyazaki-u.ac.jp). For example, S. pombe *ribosomal protein L32 (Rpl32) paralogs are encoded by two paralogous genes, rpl32-1* (SPBC16C6.11) and *rpl32-2* (SPAC3H5.10). These ribosomal protein paralogs have many common and distinct properties, such as (a) a very similar amino acid sequence among paralogs, (b) a high mRNA expression correlation among paralogs, and (c) the whole functional class, required the whole genomic duplication [4] or small-scale duplications [5], implying a low level of functional differentiation and possibly an mRNA dosage increase as an explanation for the retention of duplicates in ribosomal proteins [6]. However, recent studies showed that duplicated ribosomal proteins have various functional divergences [7] [8]. Komili et al. (2007) proposed that different combinations of RP paralogs even generate “ribosome codes” which are involved in translational regulation of specific mRNAs [9]–[11].

An essential function of ribosomal proteins is to interact with rRNA to constitute protein synthesis machinery- ribosomes [12]. Whereas many studies have revealed that some ribosomal proteins have “extraribosomal functions” [10] [13]–[17]. Our lab reported previously that Rpl32-2 specifically bound to DNA sequence containing GTTGGT, activating transcription of reporter genes in GAL4-base hybrid system in *S. cerevisiae* [18]. Recently, we have reported that deletion of Rpl32 paralogs causes reduction of ribosome level which may trigger flocculation of fission yeast cells [19]. In the present study, we further report that Rpl32 paralogs, Rpl32-1 and Rpl32-2 are involved differently in the regulation of the transition between proliferation and quiescence which are two distinct cell states for all organisms. When the nutrient is sufficient, yeast cells are normally in proliferation state, with high metabolism rate and active cell division, while under nutrition deprivation conditions such as a stationary phase culture, cells are usually in quiescence state with low metabolism rate and inactivated division, as well as high resistance to environment stress [20]. Yeast cells can shift between proliferation and quiescence in response to environmental cues. This work focuses on the functional divergence of Rpl32 paralogs in proliferation and the transition from proliferation to quiescence, as well as their molecular mechanisms.

## Results

### Rpl32-1 and Rpl32-2 Expressed Distinctively in Fission Yeast Cells in Proliferation or Proliferation to Quiescence Transition

**Figure 1:**
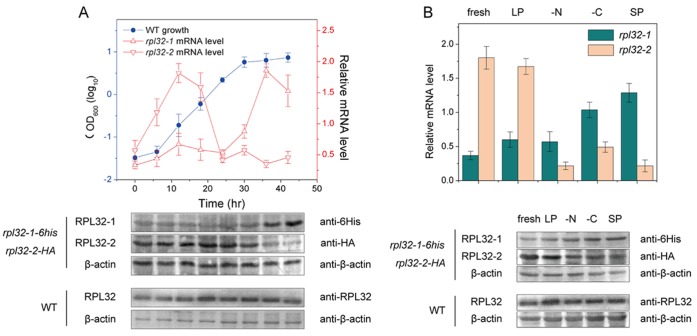
Expression of Rpl32 paralogs varied with different nutrient conditions during cultivation. (A) The growth curve of WT cells (upper panel), and changes of mRNA level (upper panel) and protein level (lower panel) of Rpl32 paralogs in WT cells during cultivation. (B) mRNA level (upper panel) and protein level (lower panel) of Rpl32 paralogs in WT cells cultured in fresh EMM2, cell-free SP EMM2, cell-free LP EMM2, EMM2-N or EMM2-C medium respectively. QPCR was used for analysis of transcription level standardized with *ACT1*. Western blot was used for analysis of protein level and β-actin was an internal control.

As shown in Fig. 1A upper panel, during log phase fission cells expressed *rpl32-1* at a lower basic level while expressed *rpl32-2* highly. The *rpl32-2* expression reached to a peak level before mid-log phase and then slowed down to a lower basic level before end of log phase. In contrast, when fission yeast cells were entering into stationary phase they expressed *rpl32-2* at a lower basic level while raised expression of *rpl32-1* rapidly. The *rpl32-1* expression reached to a peak level when cells just entered into stationary phase (at ∼36 h, early stationary phase). To further confirm differential expression patterns of these two paralogs during the course of cell growth, we constructed double-labeled mutant strain *rpl32-1-6his-rpl32-2-HA*. Western blot on *rpl32-1-6his-rpl32-2-HA* cells respectively using antibodies against 6His or HA also confirmed that in log phase Rpl32-2 was highly expressed and Rpl32-1 was lowly expressed in cells; in contrast, in early stationary phase protein level of Rpl32-1 was upregulated and Rpl32-2 was downregulated (Fig. 1A, lower panel). Since heterogeneous molecular weight of Rpl32-1-6His and Rpl32-2-HA, the Rpl32-2 antibodies against Rpl32 paralogous proteins was used for Western Blot on Rpl32 in unlabeled WT cells and results showed that total protein of Rpl32 remained at the same level in WT cells in both log phase and early stationary phase (Fig. 1A lower panel).

We hypothesize that after exponential growth, changes of expression patterns of Rpl32 paralogous genes are related with need for cells to adjust metabolism status to transit from proliferation to quiescence state when cells sense a shortage of nutrients in the medium [21]–[24]. Since cells can be induced to enter into quiescence state by nitrogen stress, carbon stress or stationary phase culturing [20], proliferating cells in log phase were transferred into fresh EMM2, cell-free log phase medium (LP), cell-free stationary phase medium (SP), nitrogen deficient EMM2-N medium and carbon deficient EMM2-C medium for further cultivation for 12 h. QPCR analysis showed that in cells grown in rich media such as fresh EMM2 and LP, *rpl32-2* mRNA level was higher than *rpl32-1*, whereas, in cells cultured in SP, EMM2-N and EMM2-C, the mRNA levels of *rpl32-2* and *rpl32-1* were in reverse (Fig. 1B upper panels). Western blot on *rpl32-1-6his-rpl32-2-HA* cells grown in above various media confirmed similar expression patterns of Rpl32-2 paralogs at protein level to mRNA level. However, the total protein expression level of Rpl32 stayed nearly at the same level in WT cells grown in all tested media (Fig. 1B lower panel).

### There is only a Single Amino Acid Difference at 95^th^ Position between the Mature Protein Rpl32-1 and Rpl32-2

**Figure 2:**
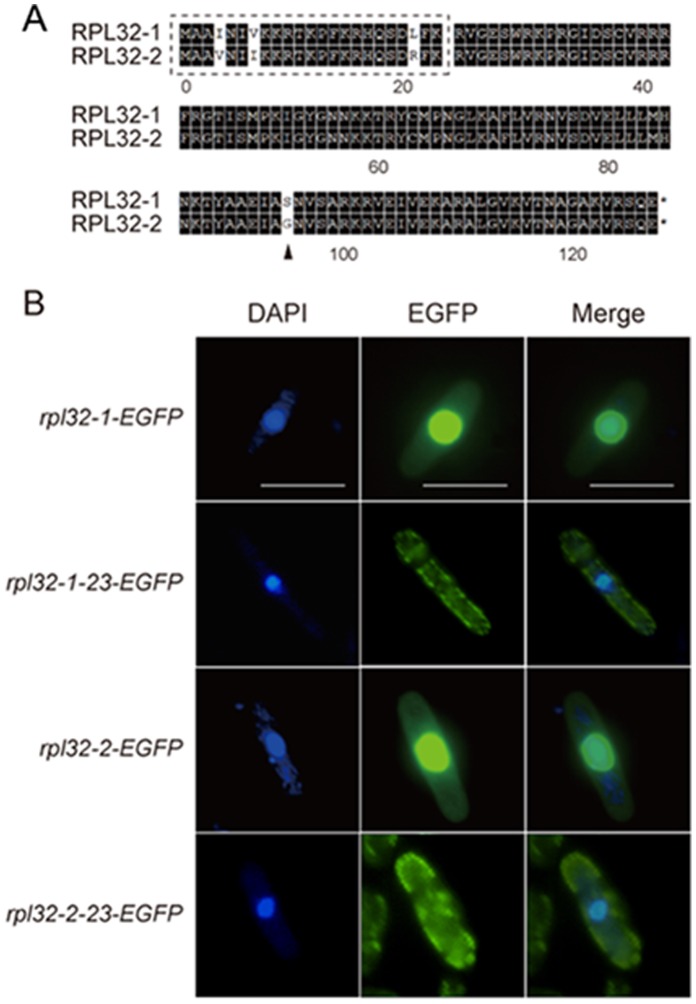
Clustal alignment and nuclear localization signal sequence analysis of Rpl32 paralogs. (A) Clustal alignment between Rrpl32-1 and Rpl32-2. Identical residues shared by these two paralogs are shaded black. (B) Localization of DAPI fluoresence (left), EGFP fluoresence (middled) and merged fluoresece (right) in *rpl32-1-egfp*,*rpl32-2-egfp*, *rpl32-1-23-egfp* or *rpl32-2-23-egfp* cells in log phase. Scale bar: 10 µm.

Alignment between Rpl32-1 and Rpl32-2 in *S. pombe* (Fig. 2A) shows that they share 96.85% similarity in amino acids sequence. There are only 4 different residues, Ile 4 Val, Val 7 Ile, Leu 21 Arg and Ser 95 Gly, in 127 amino acids of Rpl32 paralogs [5]. It is known that after ribosome proteins are synthesized in the cytosol, they need to enter into the nucleus to be assembled into ribosomal subunits, and then move back to the cytosol to assist protein synthesis [25] [26]. Analysis by PSORT II software for recognition of signal sequence (http://psort.hgc.jp/form2.html) suggests that the first 23 amino acids of Rpl32-1 or Rpl32-2 may be a nuclear localization signal sequence (Fig.2A). Thus, we constructed mutants harboring overexpression plasmids with a gene, respectively, coding Rpl32 paralogs or Rpl32 paralogs deleted of nuclear localization signal peptide, all of which were labeled with EGFP. Fluorescent microscopic images showed that Rpl32-1-EGFP and Rpl32-2-EGFP were located in the nucleus, while N-terminal 23 amino acids lacking Rpl32-1-23-EGFP and Rpl32-2-23-EGFP were found in the cytosol (Fig. 2B). This result confirmed the function of 1–23 amino acids as a nuclear localization signal sequence. In mature Rpl32 proteins without nuclear localization signal sequence, the only different amino acid between two paralogs is Ser 95 Gly. So a site-directed mutagenesis method was used to replace Ser 95 on Rpl32-1 with Gly and Gly 95 on Rpl32-2 with Ser to create Rpl32-1M and Rpl32-2M mutant proteins, respectively, for determination of their functional differences in the following experiments.

### Overexpression or Deletion of Rpl32-1 or Rpl32-2 Exerted Different Effects on Cell Growth

**Figure 3:**
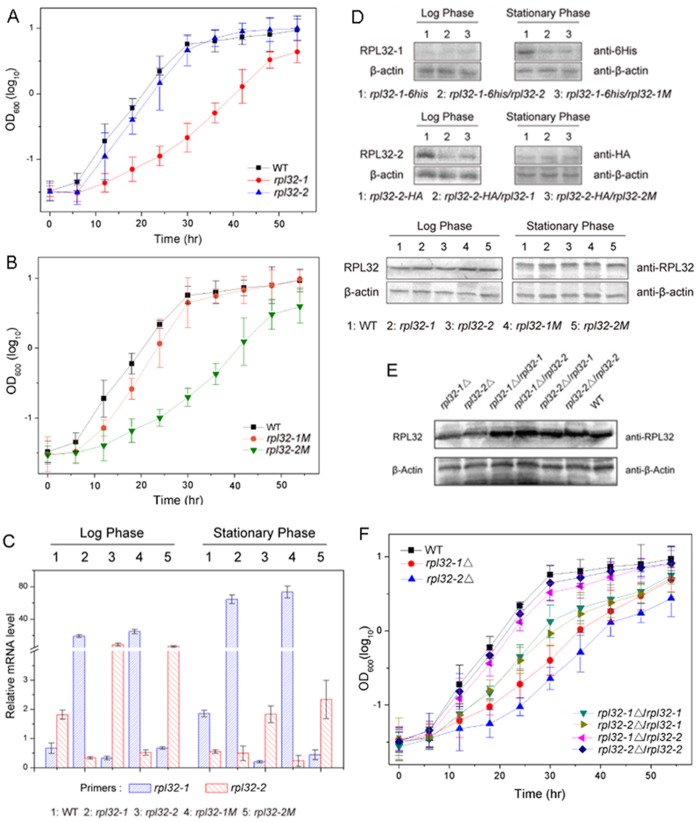
Overexpression or deletion of Rpl32 paralogs affected cell growth and their counterpart expression. (A) Growth curve of WT, *rpl32-1* or *rpl32-2* cells. (B) Growth curve of WT, *rpl32-1M* or *rpl32-2M* cells. (C) mRNA level of Rpl32 paralogous genes in WT, *rpl32-1*, *rpl32-2*, *rpl32-1M* or *rpl32-2M* cells in log or early stationary phase, tested by QPCR standardized with *ACT1*. (D) Protein level of Rpl32-1 in *rpl32-1-6his*, *rpl32-1-6his/rpl32-2* or *rpl32-1-6his/rpl32-1M* cells, Rpl32-2 in *rpl32-2-HA*, *rpl32-2-HA*/*rpl32-1* or *rpl32-1-6his/rpl32-2M* cells, and total Rpl32 in WT, *rpl32-1, rpl32-2, rpl32-1M* or *rpl32-2M* cells in log or stationary phase, tested by Western blot. β-actin was used as internal control. (E) Protein level of total Rpl32 in *rpl32-1*△, *rpl32-2*△, *rpl32-1*△*/rpl32-1, rpl32-1*△*/rpl32-2, rpl32-2*△*/rpl32-1, rpl32-2*△*/rpl32-2,* or WT cells in log phase, tested by Western blot. β-actin was used as internal control. (F) Growth curve of *rpl32-1*△, *rpl32-2*△, *rpl32-1*△*/rpl32-1, rpl32-1*△*/rpl32-2, rpl32-2*△*/rpl32-1, rpl32-2*△*/rpl32-2*, or WT cells.

We constructed mutants harboring plasmid pREP3X overexpressing Rpl32-1, or Rpl32-2, or Rpl32-1M, or Rpl32-2M, respectively, in order to mimic the expression pattern of Rpl32 paralogs for determination of their effects on cell growth states. The growth curves of *rpl32-1* and *rpl32-2* cells showed that Rpl32-1 overexpression slowed down apparently cell growth while Rpl32-2 overexpression had no significant effect on it, compared to that of WT cells (Fig. 3A). Interestingly, like Rpl32-1, Rpl32-2M overexpression inhibited cell growth, while, like Rpl32-2, Rpl32-1M overexpression did not exert effect on cell growth, suggesting the ability of Rpl32-1 overexpression to cause growth defects is mediated by serine-95. QPCR analysis (Fig. 3C) showed that as compared to WT cells in log phase, *rpl32-1* mRNA level increased by 40 times and *rpl32-2* mRNA level decreased by 81.5% in *rpl32-1* cells, while *rpl32-1* mRNA level decreased by 50% and *rpl32-2* mRNA level increased by 4.5 time in *rpl32-2* cells; as compared to WT cells in early stationary phase, *rpl32-1* mRNA level increased by 30 times and *rpl32-2* mRNA level decreased only by 10% in *rpl32-1*cells, while *rpl32-1* mRNA level decreased by 90% and *rpl32-2* mRNA level increased by 3 times in *rpl32-2* cells. Apparently, overexpression of either *rpl32-1* or *rpl32-2* reduced mRNA level of its paralog, whereas overexpression of *rpl32-2* reduced mRNA level of *rpl32-1* less in *rpl32-2* cells in log phase than in early stationary phase and overexpression of *rpl32-1* reduced mRNA level of *rpl32-2* less in *rpl32-1* cells in early stationary phase than in log phase. It is notable that *rpl32-1M* mRNA level in *rpl32-1M* cells was similar to *rpl32-1*mRNA level in *rpl32-1* cells, and *rpl32-2M* mRNA level in *rpl32-2M* cells was similar to *rpl32-2* mRNA level in *rpl32-2* cells, implying that the single site mutagenesis did not change the mRNA level of corresponding *rpl32* paralogs. Therefore we can exclude the possibility that differential growth phenotypes reported above were resulted from different transcript levels of either *rpl32* paralog rather than 95^th^ amino acid. Western blot showed that protein expression level of Rpl32-1 was decreased in *rpl32-1-6his/rpl32-2* and *rpl32-1-6his/rpl32-1M* cells as compared to *rpl32-1-6his* cells harboring empty plasmid (Fig. 3D upper panel) and protein expression level of Rpl32-2 was reduced in *rpl32-2-HA/rpl32-1* and *rpl32-2-HA/rpl32-2M* cells compared to *rpl32-2-HA* cells harboring empty plasmid (Fig. 3D middle panel), whereas total protein level of Rpl32 were almost unchanged in all of *rpl32-1*, *rpl32-2*, *rpl32-1M*, and *rpl32-2M* cells as compared to WT cells (Fig. 3D lower panel), implying that expression of Rpl32 paralogs is also regulated at translational level, and the protein expression level of corresponding Rpl32 paralogs was not influenced by the single site mutation at 95^th^ position.

Gene deletion experiments showed that in comparison with WT cells, total protein level of Rpl32 paralogs decreased significantly in *rpl32-1*△ or *rpl32-2*△ deletion cells while remained unchanged in four mutants rescued by reintroducing Rpl32 paralogs on the plasmid pREP3X, *rpl32-1*△/*rpl32-1*, *rpl32-1*△/*rpl32-2*, *rpl32-2*△/*rpl32-1* and *rpl32-2*△*/rpl32-2* (Fig. 3E), suggesting that reduced expression of Rpl32 paralogs caused by deleting *rpl32-1* or *rpl32-2* could be complemented completely by its paralog and these paralogs had “dosage benefit” [27]. Growth curve analysis (Fig. 3F) illustrated that deleting either *rpl32* paralog could impair cell growth; especially the growth defect was more severe in *rpl32-2*△ deletion cells than *rpl32-1*△ deletion cells. However, from the growth curve (Fig. 3F), we can see that reintroduction of Rpl32-2 almost could restore normal growth to either *rpl32-1*△ or *rpl32-2*△ deletion mutant, but reintroducing Rpl32-1 only partially rescued the growth defect of *rpl32-1*△ or *rpl32-2*△ deletion mutant.

### Cellular Analysis Explores the Physiological Significance and Mechanism of Differential Expression of Rpl32-1 and Rpl32-2

In order to elucidate the physiological significance and mechanism of Rpl32 paralogs differential expression, we compared phenotypes between WT and Rpl32 paralogs overexpression cells in different growth states.

**Figure 4:**
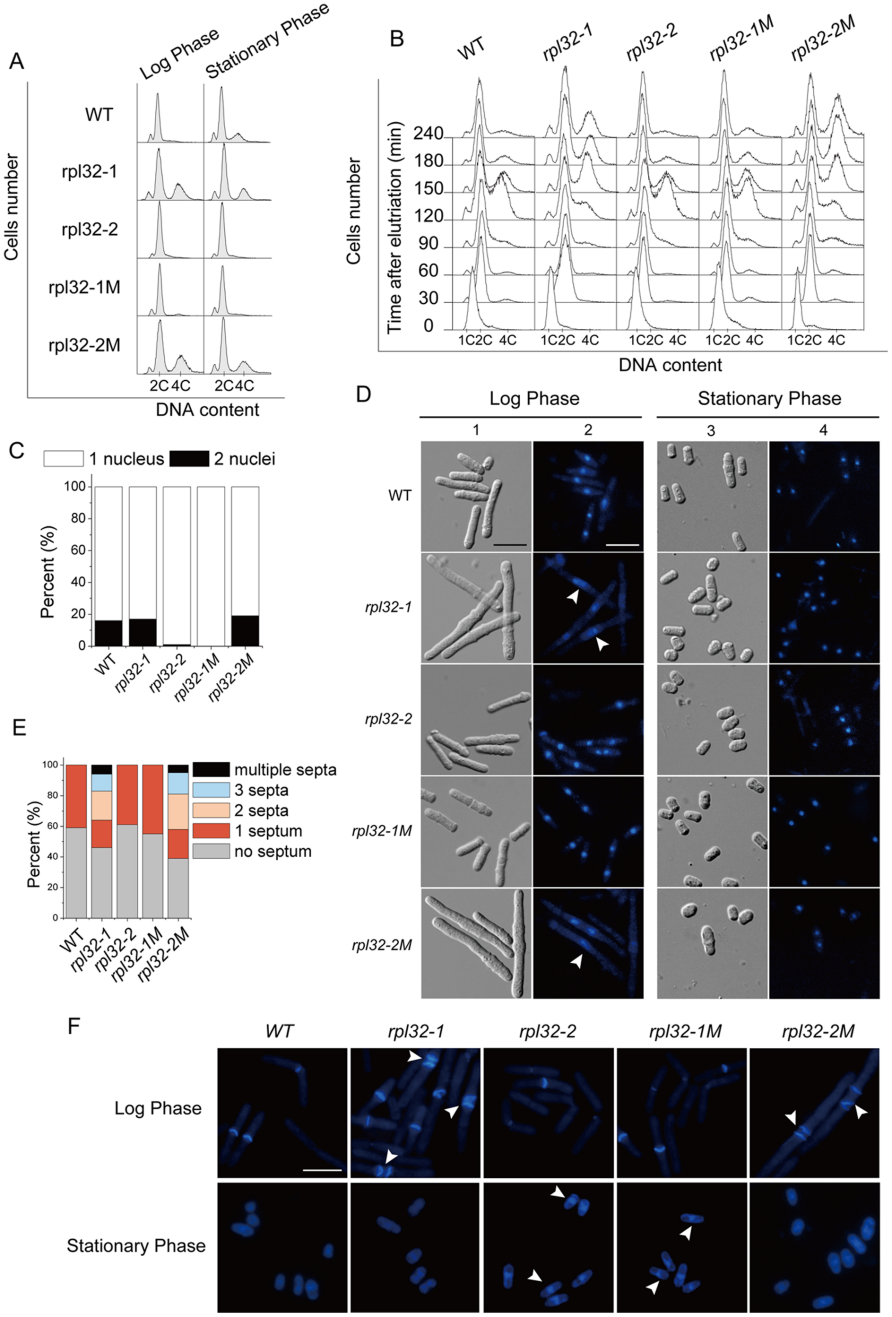
Cellular analysis of cells overexpressing Rpl32 paralogs. (A) DNA component in *rpl32-1, rpl32-2*, *rpl32-1M, rpl32-2M*, or WT cells in log or stationary phase, tested by FACS. 2C indicates the DNA content in a cell containing one G2 nucleus or a mitotic cell containing two G1 nuclei. 4C indicates the DNA content in a cell containing two G2 nuclei. (B) DIC and DAPI images of cells in (A) in log or stationary phase. Arrow indicates defect in nuclei division. scale bar: 10 µm. (C) Nuclei counting of cells from (B) in stationary phase. 300 cells per sample were counted. (D) CW images of septa in cells from (A) in log phase and stationary phase. Arrow indicates multiple septa or septa-like stucture. Scale bar: 10 µm. (E) Quantification of the percentage of septa in cells in (D) in log phase. 300 cells per sample were counted. (F) DNA component in cells synchronized with HU in (A) duiring elutriation, analysed with FACS.

FACS analysis (Fig. 4A) showed that in log phase WT cells exist essentially as 2c DNA cells; in early stationary phase many cells contained 4c DNA which reflected retardation of their cell separation after nucleus division. Interestingly, in log phase *rpl32-1* cell population had many 4c DNA cells as seen in WT cells in early stationary phase, while in early stationary phase *rpl32-2* cells were not found to contain 4c DNA, implying that highly expressed Rpl32-1 inhibited cell separation and prolonged cell separation time while high expressed Rpl32-2 promoted cell separation. Elutriation of cells which were previously synchronized at G1/S phase with HU (Fig. 4B) showed that cells from all strains doubled their DNA and entered into the G2 phase after 30 min of elutriation, indicating null effect of highly expressed Rpl32-1 or Rpl32-2 on DNA duplication. After 2 h of elutriation WT and *rpl32-2* cells showed significant population with 4c DNA, while cells with 4c DNA occurred less in *rpl32-1* cell populations, implying that nucleus division in *rpl32-1* cells was retarded [28]. After 4 h of elutriation, WT and *rpl32-2* cells essentially had no 4c DNA, while many *rpl32-1* cells stayed still in the stage with 4c DNA. These results suggested that high expression of Rpl32-1inhibits nucleus division and cell separation.

DIC (differential interference contrast microscope) observation showed that in log phase, WT cells were rod-like in morphology, *rpl32-2* cells had no significant morphological difference from WT cells, while *rpl32-1* cells were longer equivalent to approximately 4× the length of WT cells (Fig. 4D1); in early stationary phase, all of *rpl32-2* cells, and most of WT and *rpl32-1* cells showed the smaller pear-like shape, but some of WT and *rpl32-1* cells exhibited a little longer pear-like shape (Fig. 4D3). Thus, DIC results also suggest that highly expressed Rpl32-1 suppressed cell separation while highly expressed Rpl32-2 promoted cell separation.

DAPI nucleus staining showed that in log phase smaller mononuclear morphology of *rpl32-2* cells was not different from WT cells, while *rpl32-1* cells often exhibited a larger nucleus which reflected interruption of nucleus division (Fig. 4D2), consisted with 4c DNA cells explored by FACS analysis. In early stationary phase, 16% of WT and *rpl32-1* cells contained two nuclei, while *rpl32-2* cells with doubled nuclei were seldom found (Fig. 4D4), consistent with DIC data.

CW (calcofluor white) staining (Fig. 4E, F) explored that in log phase both WT and *rpl32-2* septum positive cells had only one septum each, while 66.7% *rpl32-1* septum positive cells had multiple septa each, suggesting that highly expressed Rpl32-1 inhibited cell separation, resulting in accumulation of septa. In early stationary phase, the nutrient stress withheld cells from reaching division threshold in size, so cells became smaller and formed no septa (Fig. 4F). Interestingly, in early stationary phase, though some cells of WT and *rpl32-1* had a little larger size and two nuclei they lacked septum, while some *rpl32-2* cells exhibited septum-like structure though they were smaller (Fig. 4D), indicating a promotive role of Rpl32-2 on formation of cell septum.

It is notable that *rpl32-1M* mutant cells shared the same cellular phenotype with *rpl32-2* cells, and *rpl32-2M* mutant cells shared the same cellular phenotype with *rpl32-1* cells (Fig. 4A–F), again, indicating that the functional differences of Rpl32 paralogs are determined by their difference of the 95^th^ amino acid.

### Microarray Analysis Explores some Genes Regulated Distinctively by Rpl32-1 and Rpl32-2

**Figure 5:**
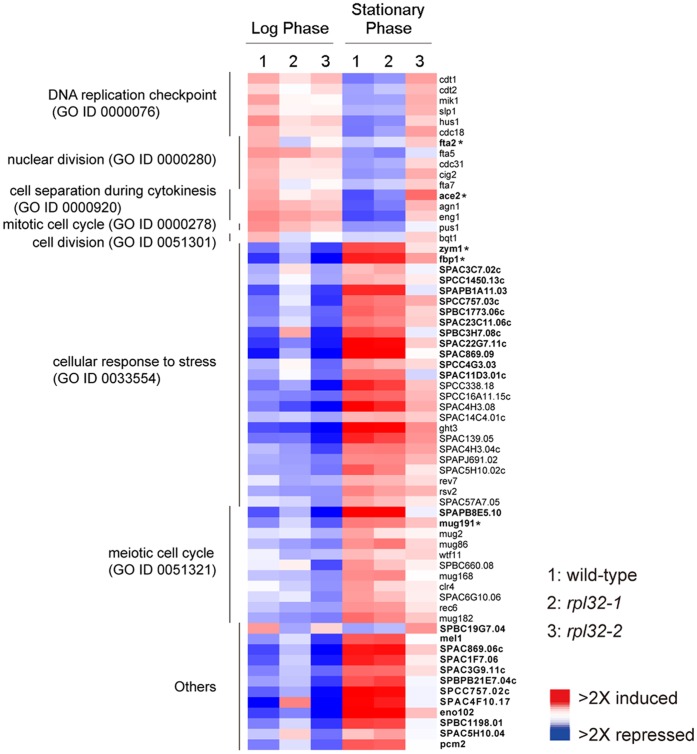
Transcriptomic patterns of WT, *rpl32-1* or *rpl32-2* cells respectively in log or stationary phase. Color panel indicates relative increase (red), decrease (blue) and median (white) of transcription level for 64 genes regulated by Rpl32-1 and Rpl32-2 in a distinctive way (at least 2-fold changes). Bold type means genes are regulated oppositely by both Rpl32 paralogs (Fold change >2) and nonbold type means genes are regulated by either of Rpl32 paralogs (Fold change >2). * Changes in transcripts level were confirmed by QPCR.

A microarray analysis was used to identify downstream genes regulated by Rpl32 paralogs and the transcription fold change >2 was designated as the significant change. We found that 29 genes were regulated oppositely by highly expressed Rpl32-1 and Rpl32-2, and other 35 genes were significantly regulated by one of Rpl32 paralogs, but not by the other (Fig. 5). Above 64 genes regulated distinctly by Rpl32-1 and Rpl32-2 in cells in log phase and early stationary phase were used to create a comparative transcriptiomic pattern (Fig. 5). As shown in Fig. 5, some genes were highly transcribed and the others were lowly transcribed in WT cells in log phase as compared to early stationary phase, while transcription levels of these genes were reversed in WT cells in early stationary phase as compared to log phase. Interestingly, the transcription pattern of *rpl32-1* cells in log phase was different from WT cells in log phase, but was similar to WT cells in early stationary phase, i. e., some genes expressed highly in WT cells were downregulated in *rpl32-1* cells while some genes expressed lowly in WT cells were upregulated in *rpl32-1* cells; in contrast, the transcription pattern of *rpl32-2* cells in early stationary phase was not identical to WT cells in the same phase, but was similar to WT cells in log phase, supporting that highly expression of Rpl32-2 is favor for cell proliferation while Rpl32-1 for transition from proliferation to quiescence.

Significantly enriched Gene Ontology (Fig. 5), which is based on GeneDB (http://www.genedb.org/genedb/pombe/index.jsp), clearly shows that 16 genes among above 64 genes are involved in mitotic cell cycle, cell separation during cytokinesis, DNA replication and nucleus division. These genes were highly expressed in WT cells in log phase while were downregulated when cells entered into early stationary phase. Overexpression of Rpl32-2 upregulated mRNA levels of above genes in cells in early stationary phase, especially the mRNA level of *fta2,* which codes a protein crucial for segregation of the duplicated sister chromatids into two equal sets [29], and *ace2*, which codes a protein crucial to cell separation [30] [31]. In contrast, overexpression of Rpl32-1 downregulated mRNA levels of above some genes, especially, apparently reduced mRNA levels of both *fta2* and *ace2* in cells in log phase. In addition to the regulation of cell proliferation, the two paralogs are also involved in the response to environmental nutrient status. Expression of 25 genes related to cellular response to stress were upregulated in WT cells in early stationary phase as compared to log phase, whereas expression of these genes were downregulated in *rpl32-2* cells in early stationary phase and were upregulated in *rpl32-1* cells in log phase. It is known that the meiotic cell cycle as a process of yeast sexual reproduction is also a cellular response to negative environments [32]. We found that expression of 12 genes involved in meiotic cell cycle were more highly expressed in stationary phase than log phase, however overexpression of Rpl32-2 downregulated transcription of these genes in stationary phase, and overexpression of Rpl32-1 upregulated transcription of these genes in log phase.

From the results of microarray analysis, we randomly determined 5 genes including *fta2* and *ace2*, with an average transcription fold change >2, which are regulated oppositely by Rpl32 paralogs. We performed QPCR analysis and confirmed that, consistent with that of microarray analysis, these genes were differentially expressed in cells in log phase and stationary phase, and upregulated or downregulated in cells overexpressing either Rpl32 paralog in different growth phases as compared to WT cells (seen noted in Fig. 5).

## Discussion

### The Two Paralogs of Ribosomal Protein L32 Function Differently in Proliferation and Quiescence by Regulation of Different Genes Expression

This study first explores that expression patterns of RPL32 paralogous genes in *S. pombe* were different from each other: Rpl32-2 had higher expression level than Rpl32-1 in log phase, while Rpl32-1 had a higher expression level than Rpl32-2 in early stationary phase. Further study found that overexpression of Rpl32-1 inhibited cell growth while overexpression of Rpl32-2 did not. Deleting Rpl32-2 impaired cell growth more severely than Rpl32-1. Cell growth impaired by deleting either paralog could be rescued completely by reintroducing Rpl32-2, but only partly by Rpl32-1. We propose that Rpl32-2 plays a leading role in proliferation, while Rpl32-1 functions in transition from proliferation to quiescence. The reason why overexpression of Rpl32-2 did not promote cell division in log phase might be due to that endogenous expression level of Rpl32-2 in log phase was already up some threshold for cell proliferation so extra overexpressed Rpl32-2 would bind to its own transcripts, regulating their splicing to modulate the protein level of Rpl32-2 itself [33] [34]. Western blot analysis also supported that overexpression of Rpl32-2 did not raise total protein level of Rpl32 in cells. Since endogenous expression level of Rpl32-2 was very low in early stationary phase, recombinant overexpressed Rpl32-2 in cells in this phase was used as a model to determine functions of Rpl32-2. For the same reason, recombinant overexpressed Rpl32-1 in cells in log phase could be used as a model to determine functions of Rpl32-1. Following this lead, we further proved that Rpl32-2 favored for cell division and septum formation, while Rpl32-1 suppressed nucleus division and cell separation. Previous research work reported that the hyper-active SIN (septation initiation network) pathway also could cause multiple septa formation [35]. If the SIN pathway was activated in asynchronous cells, the cells stopped polarized growth and did not elongate, arresting as mononucleate or binucleate cells with multiple septa [36]–[38]. But in our observations, *rpl-32-1* cells with multiple septa were much longer than WT cells so the elongation seemed not to be ceased. And the transcriptomic data (NCBI Series Entry: GSE43827) also shows that the expression profiles of genes related to SIN pathway have no difference between WT and Rpl32 paralogs overexpression cells. So we believe that the multiple septa we observed are more likely to be resulted from the suppression of cell separation by Rpl32-1 rather than the hyper-activation of SIN pathway.

Transcriptomics analysis suggested a molecular mechanism based on which Rpl32 paralogs function distinctly in proliferation and transition from proliferation to quiescence by regulation of expression of a subset of genes related with cell division and stress response in a distinctive way. First, compared to WT cells, overexpression of Rpl32-2 promoted expression of some genes related to mitotic cell cycle, cell separation and nuclear division, such as *ace2* and *fta2*, in *rpl32-2* cells in early stationary phase while overexpression of Rpl32-1 inhibited expression of *ace2* and *fta2* in *rpl32-1* cells in log phase. Second, overexpression of Rpl32-2 downregulated expression of many genes related to cellular responses to environmental nutrient stress in *rpl32-2* cells in early stationary phase, while overexpression of Rpl32-1 upregulated expression of these genes in *rpl32-1* cells in log phase, compared to WT cells, which is consistent with that transition from proliferation to quiescence is a cellular response to stress. Third, high expression of Rpl32-2 also downregulated, while high expression of Rpl32-1 upregulated those genes involved in meiotic cell cycle which is also one kind of the responses to environmental stress [32], compared to WT cells. The study concerning how Rpl32 paralogs regulate their target gens expression is in proceeding, and before it is elucidated we may not exclude the possibility of indirect effects due to cell-cycle arrest yet.

### Expression of Proliferation and Quiescence Related Genes may be Regulated by Changing the Ratio of RPL32 Paralogs

Although Rpl32 paralogs have different functions in cell proliferation and transition from proliferation to quiescence, they do share the same basic ribosomal protein function. (1) Deletion of either of the two paralogs was not vital to cells, but the double deletion cells could not survive (Data not shown), consistent with the report by Kim et al. (2010) [33]; (2) single deletion of either paralogous gene led to the shortfall of total Rpl32 protein and this shortfall could be rescued by overexpression of either Rpl32 paralog; (3) deletion of either of the two paralogs impaired cell growth, and overexpression of Rpl32-2 could fully complement the growth defect of either *rpl32-1*△or *rpl32-2*△ deletion mutants. These suggest that Rpl32 paralogous genes are complementary on their ribosomal function, therefore, Rpl32 paralogous proteins have dosage benefit [6] [27] [34]. With regards to that overexpression of Rpl32-1 only partly complement the growth defect of either *rpl32-1*△ or *rpl32-2*△ deletion mutant, this may be due to its extraribosomal proliferation inhibition besides the basic ribosomal protein function.

**Figure 6:**
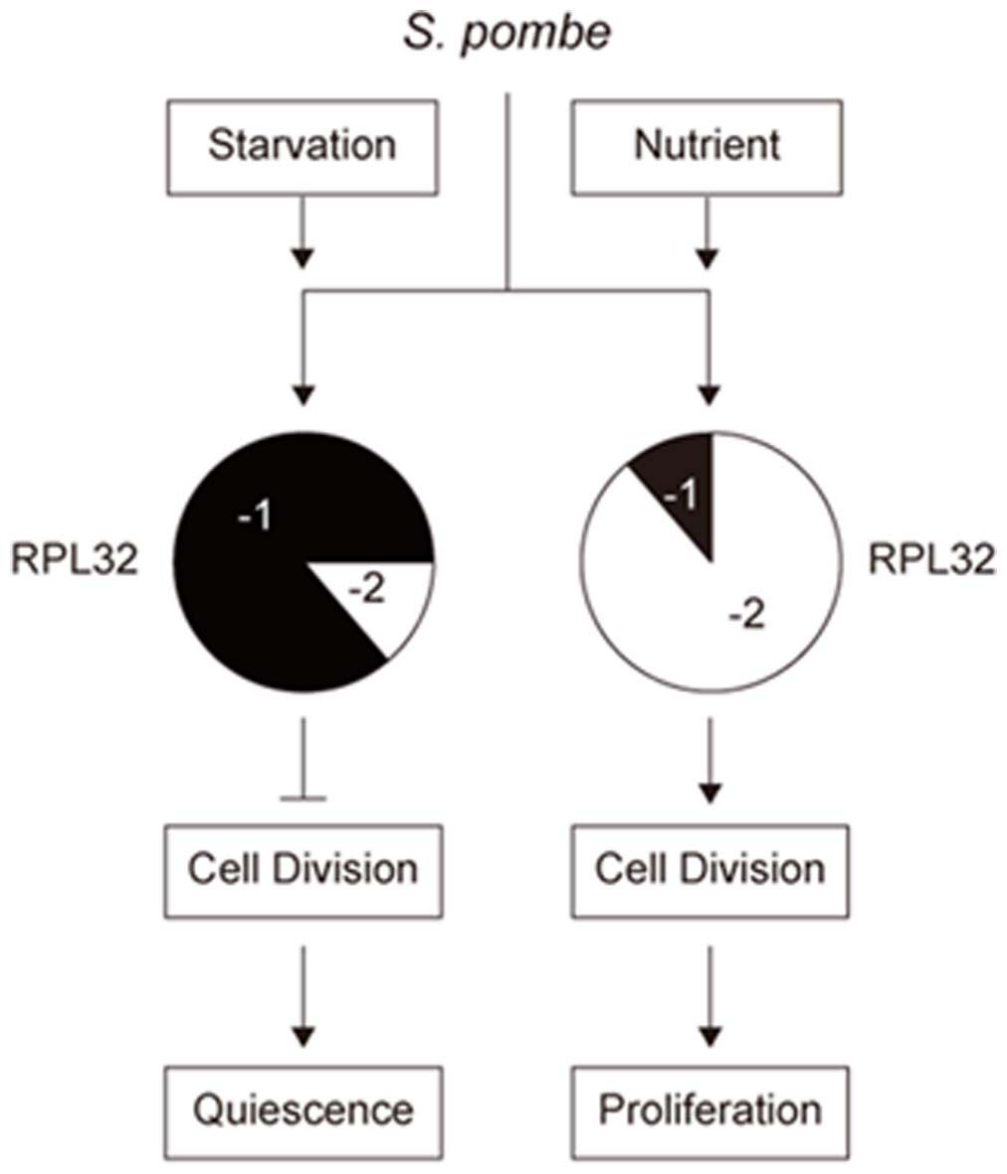
Schematic representation of a possible regulation of cell growth states by changing the ratio of Rpl32 paralogs. In rich nutrient environment, cells highly express Rpl32-2 while lowly express Rpl32-1 to promote cell division for proliferation. Nutrition deficiency induces cells to upregulate Rpl32-1 expression and downregulate Rpl32-2 expression to inhibit cell division for quiescence.

In other hand, overexpression of either paralog did not lead to accumulation of total Rpl32 protein. This suggests *S. pombe* Rpl32 has a self-feedback inhibition, as did by Rpl30 in *S. cerevisiae* [39]–[41]. In *S. cerevisiae* Rpl30 can regulate the splicing and the subsequent translation of its own mRNA, and the pre-rRNA processing to control expression of itself [42]. However, unlike *S. cerevisiae* Rpl30, *S. pombe* Rpl32 has two paralogs. We have proved that overexpression of Rpl32-1 inhibited expression of Rpl32-2 and *vice versa* in order to maintain the total RPL32 protein level unchanged. Since the change of Rpl32-1 and Rpl32-2 relative expression level had a distinctive regulation to a subset of genes related to cell proliferation, it is reasonable to conclude that specific genes expression may be regulated by changing the ratio of Rpl32 paralogs, which is considered as a ribosomal code [9] [10]. This competitive inhibition between Rpl32 paralogs on their expression provides a new evidence for the transcriptional regulation with the ribosome code in *S. pombe* [43], and also in *S. cerevisiae* [11] (Fig. 6). Certainly we have not excluded the possibility that Rpl32 paralogs individually regulate cell growth when reaching up a higher level rather than as a ribosome code as proposed.

### The Functional Differences of Rpl32 Paralogs is due to One Amino Acid Difference at Position 95^th^

Although *S. pombe* Rpl32 paralogs have distinct expression patterns and functions in different cellular states, there are only 4 different amino acids in their sequence. Three of them are located within the 1–23 amino acid residues signal sequence. In mature protein (24–127 amino acids), only one amino acid residue is different: Ser 95 for RPL32-1 and Gly 95 for Rpl32-2. By replacing Ser 95 on Rpl32-1 with Gly, we generated Rpl32-1M mutant and *vice versa* we generated Rpl32-2M mutant. Cells overexpressing Rpl32-2M had the similar features observed on cells overexpressing Rpl32-1, with impaired proliferation, and inhibited nucleus division and cell separation. Cells overexpressing Rpl32-1M exhibited similar characteristics shown on cells overexpressing Rpl32-2, with promoted proliferation, nucleus division, septa formation and cell separation. This suggests that the functional difference of these two paralogues proteins mainly is due to the divergence of the 95^th^ amino acids, rather than the other 3 different residues in the 1–23 amino acid residues signal sequence.

It is thought that protein paralogs result from a small-scale duplication including single gene or segmental duplications, or from a whole-genomic duplication [4]. Protein paralogs resulting from the whole-genome duplication are more likely to share interaction partners and biological functions than smaller-scale duplicates [44]. But the whole-genome duplications did not occurred in *S. pombe* [5], different from *S. cerevisiae* which have undergone some whole-genome duplication approximately 100 mln years ago [45]. Our results provide a new evidence of the derivation of functional diversity in paralogous proteins from small-scale duplication during evolution.

## Materials and Methods

### *S. pombe* Strains, Media and Culture Conditions

Fission yeast strains used in this work are described in Table S1.

Edinburgh Minimal Medium 2 (EMM2), EMM2-N, and EMM2-C media were made as described by Su et al. (1996) [46]. The cell-free stationary phase medium (SP) and the cell-free log phase medium (LP) were made respectively from stationary phase culture and log phase culture in EMM2. Cells were removed by centrifugation and the supernatant was sterilized by vacuum filtration through 0.45-µm (pore-size) cellulose nitrate filters.

Fission yeast cells were generally cultured in EMM2 at 30°C on a shaker with 220 rpm. When necessary, cells were cultured to log phase (10^7^ cells/ml, OD_600_ 0.5), or early stationary phase (2×10^8^ cells/ml, OD_600_ 10). For the determination of nutrient effects, cells cultured in EMM2 to log phase were harvested and washed twice with phosphate-buffered saline (PBS, pH 7.4), then transferred into same volume of EMM2, EMM2-N, EMM2-C, LP, or SP for incubation. For synchronous growth [47], cells cultured in EMM2 to log phase were collected, transferred to EMM2 with 11 mM hydroxyurea (HU) and incubated for 6 h. The synchronized cells were harvested, washed 3 times with PBS (pH 7.4), and then transferred to EMM2 for elutriation.

### Gene Tagging

The rpl32-1 and rpl32-2 were tagged with sequence encoding 6His or HA, respectively at their 3′termini at their chromosomal loci. The rpl32-1-6his-nmt1-kanmx6-rpl32-1-3′flank, rpl32-2-HA-nmt1-kanmx6-rpl32-2-3′flank and rpl32-2-HA-nmt1-leu2-rpl32-2-3′flank fragments were amplified by PCR using tagging primers sets (Table S2), and transformed into wild-type (WT) Q01 cells to generate rpl32-1-6his mutant, rpl32-2HA mutant, and double tagged mutant rpl32-1-6his-rpl32-2-HA by gene replacement [48].

### Gene Deletion

The *rpl32-1* or *rpl32-2*-targeting DNA fragments containing *kanmx6* were amplified by PCR from WT cells genomic DNA and plasmid pFA6a-*kanmx6* using deletion primers sets (Table S2), and transformed into WT cells to generate deletion mutants by gene replacement [49].

### Construction of Plasmids and Tranformants

The *rpl32-1and rpl32-2* fragments were amplified by PCR using gene-specific primer sets (Table S2) and cloned into the *Xho*I/*Sma*I sites of plasmid pREP3X to create pREP3X- *rpl32-1* and pREP3X- *rpl32-2*, separately.

DNA fragments for EGFP fusion proteins were amplified by overlap PCR using fusion primers sets (Table S2), and cloned into BamHI/NdeI sites of plasmid pESP3 to generate pESP3-*rpl32-1-egfp*, pESP3-*rpl32-1-23-egfp*, pESP3-*rpl32-2-egfp*, and pESP3-*rpl32-2-23-egfp*. Rpl32-1-23-EGFP and Rpl32-2-23-EGFP mean their N-terminal leading sequence (23 amino acids) was omitted.

DNA fragments for the *rpl32-1M* mutant, in which Ser95 (TCC) was substituted for Gly95 (GGT), or *rpl32-2M* mutant, in which Gly95 (GGT) was substituted for Ser95 (TCC), were amplified by overlap PCR [50] using point-mutated primer sets (Table S2), and cloned into the *Xho*I/*Sma*I sites of plasmid pREP3X, yielding pREP3X-*rpl32-1M* and pREP3X-*rpl32-2M*.

Above plasmids or empty plasmids (as control) were transformed into WT cells or deletion mutants (in deletion-rescuing experiments) to produce transformants using LiAc method [51].

### Western Blot

Yeast cells were harvested and lysed in 20 mM Hepes with beads [52]. Equal amount of the extracted cell lysate were separated on 12.5% SDS-PAGE and transferred to Polyvinylidene-Fluoride membranes. Purified monospecific rabbit antibody specific for His-Tag (Cell Signaling), HA (Invitrogen), β-Actin (Millipore) and RPL32 (ImmunoGen) diluted 1∶5000 with PBS-T (1×PBS, 0.05% Tween-20) containing 3% BSA, were incubated with the membrane for 4 h at 37°C. After 3 times washing with PBS, the membrane was incubated with 1∶5000 diluted secondary antibody, goat anti-rabbit-HRP conjugate (Invitrogen) for 1–2 h at 37°C. The detection reagent was TMB stabilized substrate for HRP (Promega).

### Quantitative PCR (QPCR)

Total RNA was isolated from cells using TRIZOL reagent (Invitrogen), and treated with RNase-free DNase I (TaKaRa) to eliminate any genomic DNA. First-strand cDNA was synthesized from DNA-free RNA using the SuperScript III First-Strand Synthesis System (Invitrogen) and oligo-dT primers. QPCR was done using amplification mixtures containing TaKaRa SYBR Premix Ex Taq II (Tli RNaseH Plus), reverse-transcribed RNA and primers (Table S2). The *ACT1* was used to standardize mRNA level. The experiments were repeated for three biological replicates. Reactions were run on a Rotor-Gene Q 5plex HRM System (Qiagen) using a fluorescent threshold manually set to OD 0.100 for all run [53]. The Cycles Threshold (C_T_) values were used to calculate the mean fold change of the reactions via the 2^−ΔΔCT^ method [54].

### Fluorescence Microscopy

For nucleus and septa staining, cells were fixed in 70% EtOH at 4°C for 15 min, washed twice with 100 mM PBS (pH 7.4), and stained directly with 200 ng/ml DAPI (Sigma-Aldrich) or 50 µg/ml calcofluor white (CW, Sigma-Aldrich). Fluorescence and DIC images were captured with a Zeiss Axio Imager A1 microscope (Zeiss, Jena); a Plan-APOCHROMAT 63×/1.4 oil-DIC objective lens, a Chroma GFP or DAPI filter set (Brattleboro, VT), a Sensicam QE cooled digital camera system (Cooke Corp.) and a MetaMorph/MetaFluorcombination package analysis software (Universal Imaging).

### FACS Analysis

Indicated cells were collected by centrifugation and fixed overnight in 70% cold EtOH at 4°C, followed by a 2-hr incubation at 37°C with 0.1 mg/ml RNase A in 50 mM sodium citrate buffer, pH 7.0. The treated cells were collected and washed, then resuspended in 2.5 mg/ml propidium iodide in 50 mM Na citrate buffer, pH 7.0, at 2×10^6^ cells/ml, followed by sonicating for 45s for staining. The DNA content was analyzed on Becton-Dickinson FACSCalibur™ and BD *CellQuest*™ *Pro* software [55].

### Microarray Analysis

Indicated cells cultured to their log phase or early stationary phase were pelleted by centrifugation, and immediately frozen in liquid nitrogen. Total RNA isolation, reverse-transcription and synthesis of cDNA, and microarray analysis were carried out by Gene-Tech Company Limited (Shanghai, China) as a fee-based service. GeneChip Yeast Genome 2.0 Array (Affymetrix) was used. Biotin labled amplified cDNA was stained with Streptavidin, R-phycoerythrin conjugate (SAPE). Microarray chips processed through the FS-450 fluidics station were scanned with the 30007 G scanner (Affymetrix) and analyzed with Partek Genomics Suite 6.5. Microarray analysis was performed on three independent biological replicates. A *t*-test was applied to detect differences in gene expression between each experimental group and control group. Two criteria were used to determine whether a gene was differentially expressed: fold change of ±2.0 and p value <0.05 using a two-tailed distribution, according to current reports [8] [23] [47]. The value of 2.0 is an accepted cut-off with statistical significance, and likely to be validated by QPCR.

## Supporting Information

Table S1 Fission yeast strains used in this study.
(DOC)

Table S2 PCR primers used in this study.
(DOC)
