# Supplementary material for: Paralogous Ribosomal Protein L32-1 and L32-2 in Fission Yeast May Function Distinctively in Cellular Proliferation and Quiescence by Changing the Ratio of Rpl32 Paralogs
Source: PLoS One. 2013 Apr 5;8(4):e60689. doi: 10.1371/journal.pone.0060689 (PMC3618328; doi:10.1371/journal.pone.0060689)
Supplement: Table S1 — Fission yeast strains used in this study. (DOC) [file pone.0060689.s001.doc]

Table S1. Fission yeast strains used in this study

| Strains | Genotype | Source |
| --- | --- | --- |
| Q01 | *h^-^, leu1-32* | Stragegene |
| Q0101 | *h^-^, leu1-32*/ *rpl32-1*:: *kanMX6* | This work |
| Q0102 | *h^-^, leu1-32*/ *rpl32-2*:: *kanMX6* | This work |
| Q01S1 | *h^-^, leu1-32*/ *rpl32-1*:: *rpl32-1-6his*-*kanMX6* | This work |
| Q01S2 | *h^-^, leu1-32*/ *rpl32-2*:: *rpl32-2-HA*-*kanMX6* | This work |
| Q01S | *h^-^, leu1-32*/ *rpl32-1*:: *rpl32-1-6his*-*kanMX6*, *rpl32-2*:: *rpl32-2-HA*-*leu2* | This work |
