# Supplementary material for: Paralogous Ribosomal Protein L32-1 and L32-2 in Fission Yeast May Function Distinctively in Cellular Proliferation and Quiescence by Changing the Ratio of Rpl32 Paralogs
Source: PLoS One. 2013 Apr 5;8(4):e60689. doi: 10.1371/journal.pone.0060689 (PMC3618328; doi:10.1371/journal.pone.0060689)
Supplement: Table S2 — PCR primers used in this study. (DOC) [file pone.0060689.s002.doc]

**Table S2. PCR primers used in this study**

| Primer | | Sequence (5’-3’) | | |  | | |
| --- | --- | --- | --- | --- | --- | --- | --- |
| **Oligonucleotide primer sequences used in gene tagging** | | | | | | | |
| *rpl32-1* gene replacement | | | | | | | |
| RPL32-1-5flankfo | | | CCAAGGTCAAGATACCAGG | | | | |
| RPL32-1-5flankre | | | TTAGTGGTGGTGGTGGTGGTGTTCTTGAGAACGAACTTTAGC | | | | |
| nmt1termfo | | | CACCACCACCACCACCACTAATAAAAGGAATGTCTCCCTTGC | | | | |
| nmt1termre | | | GGCAAGCTAAACGCATTACTAATAGAAAGGATTATTTCAC | | | | |
| kanmx6fo | | | CCTTTCTATTAGTAATGCGTTTAGCTTGCCTCGTCC | | | | |
| kanmx6re | | | GATTGGAATAGCTTTTTAAATCTGGATGGCGGCGTTAG | | | | |
| RPL32-1-3flankfo | | | CTAACGCCGCCATCCAGATTTAAAAAGCTATTCCAATCTCTC | | | | |
| RPL32-1-3flankre | | | GTTCGTTTAAATAACATTGAAGC | | | | |
| *rpl32-2* gene replacement | | | | | | |  |
| RPL32-2-5flankfo | | | GACAAGATGAGGTTTGAAGTTATAAG | | | | |
| RPL32-2-5flankre | | | CTAGAAGTTCTCCTCGACATTAGAATCACGACTTAGTACAG | | | | |
| leu2fo | | | CTGTACTAAGTCGTGATTCTAATGTCGAGGAGAACTTCTAGTATATC | | | | |
| leu2re | | | CCTTTCTATTAGTAATGCGTCGATCGACTACGTCG | | | | |
| nmt1termfo | | | CGACGTAGTCGATCGACGCATTACTAATAGAAAGGATTATTTC | | | | |
| nmt1termre | | | TATCCTTACGACGTGCCTGACTACGCCTAATAAAAGGAATGTCTCCCTTGC | | | | |
| RPL32-1-3flankfo | | | TTAGGCGTAGTCAGGCACGTCGTAAGGATACTCCTGAGAGCGAACC | | | | |
| RPL32-1-3flankre | | | CAGCGAGAGCGGTAGTC | | | | |
| kanmx6fo | | | CTGTACTAAGTCGTGATTCTAATCTGGATGGCGGCGTTAGTATC | | | | |
| kanmx6re | | | CCTTTCTATTAGTAATGCGTTTAGCTTGCCTCGTCCC | | | | |
| **Oligonucleotide primer sequences used in gene deletion.** | | | | | | | |
| *rpl32-1* gene deletion | | | | | | | |
| RPL32-1-5flankfo | | | GTGCTGTATTGGTTTCATATTTTAATAAAG | | | | |
| RPL32-1-5flankre | | | GCAAGCTAAACAGATCTGTTTTCGACCGTTGC | | | | |
| RPL32-1-3flankfo | | | CGCCATCCAGTTTAAAATTTAAAAAGCTATTC | | | | |
| RPL32-1-3flankre | | | GTTCGTTTAAATAACATTGAAGCTCAAG | | | | |
| *rpl32-2* gene deletion | | | |  | | | |
| RPL32-2-5flankfo | | | CAGCGAGAGCGGTAGTCGCTTGAC | | | | |
| RPL32-2-5flankre | | | GCAAGCTAAACAGATCTTTCTGTTGTGTGGGTG | | | | |
| RPL32-2-3flankfo | | | AAAAATATACAATGTAACCAAGGATG | | | | |
| RPL32-2-3flankre | | | CGCCATCCAGTTTAAAATTAGAATCACGACTTAG | | | | |
| **Oligonucleotide primer sequences used in gene overexpression and complement** | | | | | | | |
| RPL32-1fo | | | CCGCTCGAGATGGCTGCAATCAACATTGTC | | | *Xho*I | |
| RPL32-1re | | | TCCCCCGGGTTATTCTTGAGAACGAACTTTAGCG | | | *Sma*I | |
| RPL32-2fo | | | CCGCTCGAGATGGCTGCTGTCAATATCATC | | | *Xho*I | |
| RPL32-2re | | | TCCCCCGGGTTACTCCTGAGAGCGAACC | | | *Sma*I | |
| **Identification of the RPL32 nuclear localization signal sequence.** | | | | | | | |
| RPL32-1fo | GGAATTCCATATGATGAGCAAGGGCGAGGAGC | | | | | *Nde* I | |
| RPL32-1N23fo | ATCCCATATGGTGTTGGTGAATCATGGAGG | | | | | *Nde* I | |
| RPL32-1re | GAACTCTCCTGATCCTGCTGCTGATCCTGCTGATCCCTTGTACAGCTCGTCCATGC | | | | |  | |
| EGFP-1fo | GGATCAGCAGGATCAGCAGCAGGATCAGGAGAGTTCATGGCTGCAATCAACATTG | | | | |  | |
| EGFP-1re | CGCGGATCCTTATTCTTGAGAACGAAC | | | | | *Bam*H I | |
| RPL32-2fo | ATCCCATATGATGGCTGCTGTCAATATC | | | | | *Nde* I | |
| RPL32-2N23fo | ATCCCATATGCGTGTTGGAGAATCATGG | | | | | *Nde* I | |
| RPL32-2re | GAACTCTCCTGATCCTGCTGCTGATCCTGCTGATCCTTACTCCTGAGAGCG | | | | |  | |
| EGFP-2fo | GGATCAGCAGGATCAGCAGCAGGATCAGGAGAGTTCATGAGCAAGGGCGAGGAG | | | | |  | |
| EGFP-2re | GGTGGGATCCTTACTTGTACAGCTCGTCCATCG | | | | | *Bam*H I | |
| **Oligonucleotide primer sequences used in site-directed mutagenesis.** | | | | | | | |
| *rpl32-1M* (*rpl32-1 (tcc→ggt)*) | | | | | | | |
| RPL32-1Mfo | | | ATGGCTGCAATCAACATTGTC | | | |  |
| RPL32-1Mmidre | | | CAGCCGAGATTGCTGGTAATG | | | |  |
| RPL32-1Mmidfo | | | GCAGAAACATTACCAGCAATCTC | | | |  |
| RPL32-1Mre | | | TTATTCTTGAGAACGAACTTTAGC | | | |  |
| *rpl32-2M*  (*rpl32-2 (ggt→tcc)*) | | | | | | | |
| RPL32-2Mfo | | | ATGGCTGCTGTCAATATC | | | |  |
| RPL32-2Mmidre | | | GAGATTGCTTCCAACGTCTCTG | | | |  |
| RPL32-2Mmidfo | | | GACGTTGGAAGCAATCTCAGCAGC | | | |  |
| RPL32-2Mre | | | TTACTCCTGAGAGCGAACC | | | |  |
| **Oligonucleotide primer sequences used in QPCR.** | | | | | | | |
| rpl32-1fo | | GGCTGCAATCAACATTGTCAAAAAGCG | | | | |  |
| rpl32-1re | | GTTCGACATCAGAGACATTGCGAACAAG | | | | |  |
| rpl32-2fo | | GGCTGCTGTCAATATCATCAAGAAGCG | | | | |  |
| rpl32-2re | | GCTCAACATCTGAAACGTTACGGACTAG | | | | |  |
| act1fo | | GGATTCCTACGTTGGTGATGAAGCTC | | | | |  |
| act1re | | GGGTTCAAAGGAGCCTCAGTCAAC | | | | |  |
| ace2fo | | CCTCCGGAGACAATGGCTAG | | | | |  |
| ace2re | | CACAGCGATACGGACGG | | | | |  |
| fta2fo | | CATATCGCTGCAATACGTGG | | | | |  |
| fta2re | | GATTAAGCAACTTTGATGTGATGCTTAC | | | | |  |
| zym1fo | | GGAACACACTACCCAATGTAAGAGC | | | | |  |
| zym1re | | CGAAGCACATTTGCAAGAAGAGC | | | | |  |
| fbp1fo | | CGCCGATATGCACCGTACC | | | | |  |
| fbp1re | | CTCGACTTCATGTTTACTTCCAAGCC | | | | |  |
| mug191fo | | GGCTAGAGGTGCTTTGGACACC | | | | |  |
| mug191re | | CGGGACTCAACTTGTAAAGACC | | | | |  |
